# Release of β-endorphin, adrenocorticotropic hormone and cortisol in response to machine milking of dairy cows

**DOI:** 10.14202/vetworld.2015.284-289

**Published:** 2015-03-07

**Authors:** E. Fazio, P. Medica, C. Cravana, A. Ferlazzo

**Affiliations:** Department of Veterinary Sciences, University of Messina, 98168 Messina, Italy

**Keywords:** adrenocorticotropic hormone, cortisol, dairy cows, machine milking, β-endorphin

## Abstract

**Aim:**

The present study was undertaken with the objective to obtain insight into the dynamics of the release of β-endorphin, adrenocorticotrophic hormone (ACTH) and cortisol in response to machine milking in dairy cows.

**Materials and Methods:**

A total of 10 healthy multiparous lactating Italian Friesian dairy cows were used in the study. Animals were at the 4^th^-5^th^ month of pregnancy and were submitted to machine milking 2 times daily. Blood samples were collected in the morning: In baseline conditions, immediately before milking and after milking; and in the early afternoon: In baseline conditions, before milking and after milking, for 2 consecutive days. Endocrine variables were measured in duplicate, using a commercial radioimmunoassay for circulating β-endorphin and ACTH concentrations and a competitive enzyme-linked immunoassay for cortisol concentration.

**Results:**

Data obtained showed a similar biphasic cortisol secretion of lactating dairy cows, with a significant increase of cortisol concentration after morning machine milking, at both the 1^st^ and the 2^nd^ day (p<0.05), and a decrease after afternoon machine milking at the 2^nd^ day (p<0.01). One-way RM ANOVA showed significant effects of the machine milking on the cortisol changes, at both morning (f=22.96; p<0.001) and afternoon (f=15.10; p<0.01) milking, respectively. Two-way RM ANOVA showed a significant interaction between cortisol changes at the 1^st^ and the 2^nd^ day (f=7.94; p<0.0002), and between the sampling times (f=6.09; p<0.001). Conversely, no significant effects of the machine milking were observed on β-endorphin and ACTH changes, but only a moderate positive correlation (r=0.94; p<0.06) after milking stimuli.

**Conclusions:**

A wide range of cortisol concentrations reported in this study showed the complex dynamic patterns of the homeostatic mechanisms involved during machine milking in dairy cows, suggesting that β-endorphin and ACTH were not the main factors that caused the adrenocortical response to milking stimuli.

## Introduction

β-endorphin and adrenocorticotrophic hormone (ACTH) are two peptides derived from the common precursor proopiomelanocortin, which is present in the pituitary gland. A recent study showed that the ACTH secretion from melanotrophs cells in lactating rats is regulated by neuroendocrine dopamine neurons [1]. β-endorphin is involved in the endocrinological response to suckling in some animals and women, with a significant increase after 20 min, suggesting that the increased β-endorphin derives from an extra-hypophyseal source [2]. Elevated plasma β-endorphin and cortisol concentrations in unfamiliar surroundings in dairy cows were described [3]; in addition, when cows acclimate to the new surroundings, the concentrations of these hormones decrease [4]. These observations showed that endogenous opioid peptides play a role within the mechanisms causing central inhibition of milk ejection; hence, the exogenous opioid morphine inhibited both oxytocin release and milk ejection [5]. Nevertheless, the role of endogenous opioids in the regulation of milk ejection and the mechanisms of inhibition of oxytocin release in cows remains unclear [6]. In emotional stress situations, the release of oxytocin from the pituitary is inhibited with simultaneously elevated β-endorphin levels in dairy cows [7]. Moreover, a decrease of plasma β-endorphin concentrations during machine milking in cows with disturbed milk ejection and in control animals with normal milk removal were shown [8]. In addition, a recent experiment showed that β-endorphin releases was not affected by milking frequency and not correlated with the magnitude of prolactin release [9]. Furthermore, milking frequency had no effect on cortisol production [10]. Nevertheless, once-daily milking has attracted considerable research interest over the years [11].

The hypothalamo-pituitary-adrenal axis activity and circulating cortisol contents are generally considered a good index of the reaction of animals to environmental challenges [6,12], and the course of adaptation to the novel milking environment can be predicted by testing the sensitivity of the adrenal cortex to ACTH in dairy cows [6]. The increase of cortisol concentrations during milking was not likely induced by ACTH because there were no changes in ACTH concentrations [13], although the control of the adrenal glucocorticoid secretion has generally been supposed to exclusively depend on the release of ACTH [6]. The reaction towards the changeover to automatic milking system varied widely within dairy cows; hence, adaptation to this milking system was easier in animals with a higher adrenal cortex sensitivity to ACTH [14]. Increases in cortisol response to milking in the plasma, saliva and milk after ACTH administration reflected adrenal stimulation in responses to normal physiological events [15]. The relationship between cortisol and function of the mammary gland was evidenced, and cortisol seems to be important in the reduction of mammary epithelial cell tight junction leakiness in the udder of dairy cows [16,17]. Automated milking system induced higher cortisol level than a conventional tandem parlor in dairy cows, but median fecal concentrations of the cortisol metabolite dioxoandrostane were comparable [18] similarly the milk cortisol concentrations [19]. Circulating cortisol concentrations increased physiologically during normal machine milking of cows [3], conversely these concentrations decreased during suckling in dairy cows and their calves [20]. Moreover, an increased cortisol production in cows with milk ejection disorders was not obvious [21].

Since milking can represent psychophysical and emotional stimulations, with or without effects on the behavior, welfare [22-24] and metabolic status [25], aim of study was to obtain insight into the dynamics of the release of β-endorphin, ACTH and cortisol in response to machine milking in dairy cows.

## Materials and Methods

### Ethical approval

This study was performed according to the guideline recommended by the Directives 86/609/EEC and 2010/63/EU, and was approved by Ethical Committee for the Care and Use of Animals of the University of Messina.

### Dairy cows

A field study using ten multiparous, pregnant Italian herds was conducted to determine the effects of machine milking on β-endorphin, ACTH and cortisol changes. Dairy cows were five (5.2±2.54) years of age, milk production (25±5.0 kg) and lactation stage (days in milk: 110±10). All subjects approximately calving in the same period were at the 4^th^-5^th^ month of pregnancy on the basis of a pregnancy test carried out at 21 days post breeding. The milkers were familiar with the milking machine and cows were milked twice daily (06:00 a.m. and 03:30 p.m.), so data collected during this period were used to provide baseline values, at 06:30 a.m. and at 03:30 p.m. Cows were held on a farm located in Catania, eastern Sicily (37° 30’ 4” 68 N latitude; 15° 4’ 27” 12 E longitude) 380 m at sea level. The herd structure was static without cows entering and leaving the experimental animals to minimize the effect of social stress caused by the addition of new animals into a herd; for this reason no new animals were mixed into the experimental herd during the 2-day period. All dairy cows were subjected to the same husbandry procedures, had free access to pasture during the day and fed a total mixed ration available twice day after milking; diet consisted of 59% grass silage and 41% concentrate on a dry matter basis. The composition of food ration was the same for the three farms, to minimize the effect of different diet; water was available *ad libitum*.

Cows were moved to the loose housing and milked in according to normal routines in a rotary milking parlor. Prior to milking, all cows were brought into a holding area and restrained as a group.

### Sample collection

Blood samples were collected via the coccygeal vessels in the tail, in the morning: In baseline conditions, immediately before milking at 06:00 a.m., and after milking at 07:00 a.m., and in the early afternoon: In baseline conditions, before milking at 03:00 p.m., and after milking at 04:00 p.m. for 2 consecutive days. Multiple veterinarians simultaneously collected blood samples from all cows to minimize the restraint time. Immediately after exiting the milking parlor after being milked cows were moved to a holding area, and the post-milking blood sample was collected. Blood collection took <30 s per cow. All blood samples were collected at approximately the same time each day in order to minimize any possible variation due to diurnal effects.

### Sample analyzes

In order to analyze β-endorphin concentrations, after collection an aliquot of the blood samples (2.5 mL) was transferred into polypropylene tubes containing ethylenediaminetetraacetic acid (EDTA) (1 mg/mL of blood) and aprotinin (500 kallikrein inhibitor unit/mL blood, ICN Biomedicals Inc., Aurora, OH, USA) and kept at 4°C. Plasma samples were harvested after centrifugation at 3000 × *g* for 15 min at 4°C and stored at -80°C until analysis. Peptides were extracted from plasma samples with 1% trifluoroacetic acid (high-performance liquid chromatography [HPLC] grade) and eluted with 60% acetonitrile (HPLC grade) in 1% trifluoroacetic acid. Plasma β-endorphin concentrations were measured in duplicate, using a commercial radioimmunoassay (RIA) kit (Peninsula Lab. Inc., Belmont, CA, USA). The standards supplied with the kit were of human origin; nevertheless, a synthetic bovine standard was run with all assays to confirm the cross-reactivity of the antiserum. In addition, dilutions of bovine serum samples provided a superimposable curve to those humans’ standards. The hormone assay used has a range for the amount of β-endorphin detected of 3-371 pmol/L. The sensitivity of the assay β-endorphin was 5 pmol/L. The intra- and interassay coefficients of variation (CVs) were 7.0% and 15.0%, respectively.

Serum ACTH concentrations were analyzed in duplicate using a commercially available RIA kit (ELSA-ACTH, CIS-BioInternational, Gif-sur-Yvette, France). The hormone assay used has a range for the amount of ACTH detected of 0-440 pmol/L. The sensitivity of the assay ACTH was 0.44 pmol/L. The intra- and inter-assay CVs were 6.0% and 15.0%, respectively.

Total serum cortisol concentrations were analyzed in duplicate using a competitive enzyme-linked immunoassay (EIA, RADIM, Rome, Italy) by a commercial test kit and a BRIO automated analyzer (SEAC, Rome, Italy). During the first incubation, the cortisol sample competed with cortisol conjugated to horse radish peroxidase for the specific sites of the antiserum coated on the wells. Following incubation, all unbound material was removed by aspiration and washing. The enzyme activity bound to the solid phase is inversely proportional to cortisol concentration in calibrators and samples, and is made evident by incubating the wells with a chromogen solution (tetramethylbenzidine) in substrate-buffer. Colorimetric reading was carried out using a spectrophotometer at 450, 405 nm wavelength (Sirio S, SEAC, Florence, Italy). Assay sensitivity was 13.80 nmol/L. The intra- and interassay CVs were 4.0% and 6.9%, respectively.

### Statistical analysis

Data are presented as mean ± standard deviation. To determine whether machine milking stimuli had any effect, a one-way analysis of variance for repeated measures (RM ANOVA) was applied. Significant differences between before and after milking of the 1^st^ and the 2^nd^ day values were established using Student’s paired t-test. To determine the effects of interaction between the 1^st^ and the 2^nd^ day, and between the sampling times, a two-way RM ANOVA was applied. The level of significance was set at p<0.05. All calculations were performed using the PRISM package (GraphPad Software Inc., San Diego, CA, USA). The correlation among β-endorphin, ACTH and cortisol concentrations was evaluated by linear regression (*r*), calculated using Pearson’s method.

## Results

**Figure-1:**
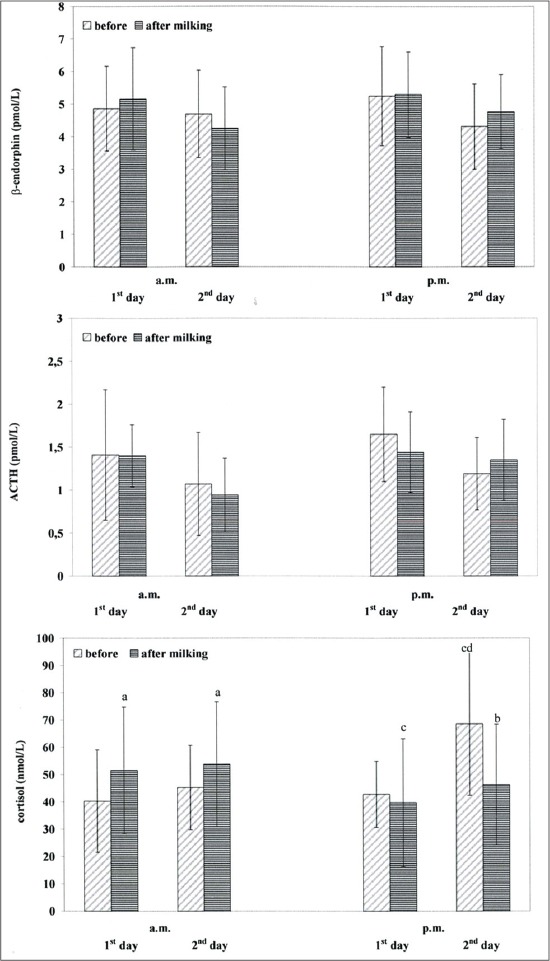
Circulating β-endorphin, adrenocorticotrophic hormone and cortisol concentrations (mean ± standard deviation) in dairy cows before and after machine milking, Different superscripts show significant differences versus before a=p<0.05; b=p<0.01; versus a.m. c=p<0.001; versus the 1^st^ day d=p<0.01

Data obtained showed that circulating β-endorphin concentrations of Italian Friesian dairy cows (Figure-1) ranged between 4.25 and 5.29 pmol/L.

Compared to baseline β-endorphin values, dairy cows showed moderate higher β-endorphin values a.m. and p.m. after machine milking at the 1^st^ day; at the 2^nd^ day moderate lower β-endorphin values a.m. after milking, and higher values p.m. after milking were observed. No significant differences were observed for the comparison between a.m. and p.m. β-endorphin values, neither between the 1^st^ and the 2^nd^ day values. One-way RM ANOVA did not show a significant effect of the treatments on the β-endorphin changes, both during a.m. and p.m. machine milking.

Data obtained showed that circulating ACTH concentrations of Italian Friesian dairy cows (Figure-1) ranged between 0.94 and 1.65 pmol/L.

Compared to baseline ACTH values, at the 1^st^ day dairy cows showed moderate baseline lower ACTH values a.m. and p.m. after machine milking; at the 2^nd^ day moderate higher ACTH values p.m. after milking were observed. No significant differences were observed for the comparison between a.m. and p.m. ACTH values, neither between the 1^st^ and the 2^nd^ day values. One-way RM ANOVA did not shown a significant effect of the treatments on the ACTH changes, both during a.m. and p.m. machine milking. A moderate positive correlation (r=0.94; p<0.06) between β-endorphin and ACTH changes after milking stimuli was obtained.

Data obtained showed that circulating cortisol concentrations of Italian Friesian dairy cows (Figure-1) ranged between 39.72 and 68.56 nmol/L.

Compared to baseline cortisol values, dairy cows showed significant higher cortisol values (p<0.05) a.m. after machine milking both at the 1^st^ and the 2^nd^ day; regarding to after p.m. milking lower cortisol values (p<0.01) at the 2^nd^ day were found, as showed by two-way RM ANOVA (f=7.94; p<0.0002). Compared to a.m. after milking, p.m. after cortisol values of the 1^st^ day were lower (p<0.01). At the 2^nd^ day, compared to a.m. baseline cortisol values, p.m. baseline cortisol values were higher (p<0.01), as showed by two-way RM ANOVA (f=6.09; p<0.001). One-way RM ANOVA showed significant effects of the machine milking on the cortisol changes, both at a.m. (f=22.96; p<0.001) and at p.m. (f=15.10; p<0.01) machine milking.

## Discussion

Circulating β-endorphin concentrations obtained in dairy cows are in accordance with data observed in dairy cows [3,4,8] values, but they were lower than data reported in previous studies in cattle [26] and lactating dairy cows [9]. The superimposable baseline β-endorphin concentration and moderate changes after milking signal that animals were unstressed both before and after milking. Therefore, dairy cows in the present study were intuitively totally accustomed to machine milking system and relaxed in their familiar surroundings.

In addition, a comparison of ACTH concentrations obtained in dairy cows with published data seem to occur within physiologically tolerable limits [5,27], but they were lower than data reported in previous studies in plasma young beef bulls [28]. Data obtained confirmed that ACTH concentrations did not change during milking, whereas cortisol increased [5]. Thus, it seems likely that no significant changes of β-endorphin and ACTH concentrations after machine milking stimuli are due to a probably unmodified pituitary gland responsiveness to milking stimuli, indicating that these hormones are not a limiting factors on the adrenocortical activity. In addition, this finding confirms concurrent regulation from the intermediate lobe, with a substantial release of both hormones from the anterior pituitary gland [29], as confirmed by positive moderate correlation between β-endorphin and ACTH changes.

Circulating cortisol concentrations obtained in dairy cows are in accordance with data reported in dairy cows [27] and bovine [30] ranges. A comparison of the results obtained in dairy cows after machine milking with published data showed a similar pattern of cortisol concentrations, with a cortisol increases after milking than baseline values [5,27].

Moreover, the highest cortisol concentrations observed in dairy cows milked during the morning, both at the 1^st^ and the 2^nd^ day could indicate that these cows were very sensitive to milking stimuli, even if all animals appeared to be calm and relaxed during milking. Overall, the magnitude of cortisol increases after morning machine milking may suggest a stimulus-response relationship and a pulsatile mode of release’s cortisol that was probably superimposed on baseline circadian variations.

Alternatively, the course of afternoon cortisol decrease observed after machine milking could be due to the possible inhibition of adrenocortical activity, reported when the presentation of stressful stimuli involved a consummatory event [31]; in this meaning, the specific consummatory event could be represented by the milk ejection induced by machine milking stimulus.

Moreover, these data confirm the cortisol daily periodicity described in milk, which was characterized by an early morning peak and a late afternoon elevation in automatic milking systems [19]. The real and concrete difficulty in determining the effect played by the machine milking method on the adrenocortical responses resulted from the fact that circulating cortisol concentrations could no reflect the existence of circadian rhythms, because minute by minute fluctuations in cortisol concentrations could be observed if more frequent blood sampling were to be employed [32]. However, this occurrence has no support in our investigations; likewise the presence or absence of a circadian rhythm cannot be ascribed to the time of year, since all blood sampling for the different groups were performed in the same time.

In addition, data obtained confirmed the evidence that the increase of cortisol concentrations during machine milking was not likely induced by ACTH because there were no significant changes in ACTH concentrations in dairy cows [5]. It seems that β-endorphin and ACTH responses were not affected by machine milking stimuli in the present study, suggesting that the HPA axis is not activated in response to machine milking, but confirming that the cortisol increase during milking under normal conditions seems to be regulated by other mechanisms than stress [33,34]. This hypothesis was confirmed by both the existence of significant interaction between cortisol changes and morning (a.m.)/afternoon (p.m.) milking, and the significant effect of sampling times (1^st^ and 2^nd^ day) on the cortisol changes.

The increases and decreases in adrenocortical responses after milking, with higher cortisol releases after morning milking compared to afternoon milking, indicated that the degree of adrenocortical activation and or inhibition was maintained, independent of the dynamics of β-endorphin and ACTH secretion. Hence, although stressful stimuli are likely to act through the HPA axis, the neuroendocrine mechanisms involved during morning and afternoon machine milking were probably different. A wide range of cortisol concentrations reported in this study showed the complex dynamic patterns of the homeostatic mechanisms involved during machine milking in dairy cows, suggesting that β-endorphin and ACTH were not the main factors that caused the adrenocortical response to milking stimuli.

## Conclusion

The interpretation of the adrenocortical responses of dairy cows to machine milking stimuli during 2 consecutive days can potentially be difficult due to the complexity of assessing an individual animal’s perception of aversive and/or predictable stimuli, as well as coping strategies and adrenocortical sensitivity can have on this response.

## Authors’ Contributions

The idea for the paper was conceived by EF and AF. The experiments were performed by PM and CC. The data were analyzed by PM and CC. The paper was written by EF and reviewed and revised by AF. All authors read and approved the final manuscript.
